# Synergistic Antinociceptive Effects of Indomethacin–Pregabalin and Meloxicam–Pregabalin in Paclitaxel-Induced Neuropathic Pain

**DOI:** 10.3390/biomedicines10061413

**Published:** 2022-06-15

**Authors:** Yurong Ma, Wenwen Liu, Lingzhi Liang, Jiaqi Ye, Chaonan Huang, Tao Zhuang, Guisen Zhang

**Affiliations:** 1 Jiangsu Key Laboratory of Marine Pharmaceutical Compound Screening, School of Pharmacy, Jiangsu Ocean University, Lianyungang 222005, China; 2019220325@jou.edu.cn (Y.M.); 2019220322@jou.edu.cn (W.L.); 2019220320@jou.edu.cn (L.L.); 2020220665@jou.edu.cn (J.Y.); 2020220623@jou.edu.cn (C.H.); 2 Jiangsu Key Laboratory of Marine Biological Resources and Environment, School of Pharmacy, Jiangsu Ocean University, Lianyungang 222005, China; 3 Systems Biology Theme, Department of Biomedical Engineering, College of Life Science and Technology, Huazhong University of Science and Technology, Wuhan 430074, China

**Keywords:** synergistic effects, neuropathic pain, inflammatory pain, pregabalin, NSAIDs

## Abstract

Neuropathic pain is often closely associated with nerve injury or inflammation, and the role of traditional nonsteroidal anti-inflammatory drugs as adjuvants for treating chemotherapy-induced peripheral neuropathic pain remains unclear. In this study, the potential synergistic antinociceptive effects of indomethacin–pregabalin and meloxicam–pregabalin were evaluated in paclitaxel-induced neuropathic pain and carrageenan-induced inflammatory pain in rodents. Although indomethacin and meloxicam alone only slightly relieved mechanical allodynia in the above two models, isobolographic analysis showed that the combination of indomethacin or meloxicam with pregabalin produced significant synergistic antinociceptive effects for paclitaxel-induced neuropathic pain (IN-PGB, experimental ED_25_ = [4.41 (3.13–5.82)] mg/kg, theoretical ED_25_ = [8.50 (6.62–10.32)] mg/kg; MEL-PGB, experimental ED_25_ = [3.96 (2.62–5.46)] mg/kg, theoretical ED_25_ = [7.52 (5.73–9.39)] mg/kg). In addition, MEL-PGB dosed via intraplantar injection into the left paw, intragastric injection, or intraperitoneal injection reversed paclitaxel-induced allodynia, indicating that they may act at multiple sites in the neuroaxis and periphery. However, indomethacin–pregabalin and meloxicam–pregabalin exerted antagonistic antiallodynic interactions in carrageenan-induced inflammatory pain in rats. Taken together, coadministration of indomethacin or meloxicam with pregabalin may possess potential therapeutic advantages for treating chemotherapy-induced neuropathic pain.

## 1. Introduction

Chemotherapy-induced peripheral neuropathy (CIPN) is a common side effect of anticancer drugs, including taxanes, platinum-based agents, and bortezomib, which severely reduces the quality of life of cancer patients [1,2,3,4,5]. Approximately two-thirds of cancer patients who receive chemotherapies may suffer CIPN within 30 days of treatment. The development of CIPN can be caused by both single and cumulative drug doses [6,7]. Paclitaxel is widely recommended as the first-line therapy for solid and blood cancers and increases both the progression-free and overall survival time of patients [8]. However, paclitaxel often induces dose-limiting peripheral neuropathic pain (allodynia, nociceptive allodynia, and spontaneous pain) in 30–78% of cancer patients [9], which may lead to discontinuation of therapy [1,10]. Hence, the growing incidence of CIPN represents a mounting clinical issue. Treating CIPN remains unsatisfactory because of the lack of fully effective analgesics [11,12]. Most of the currently available first-line drugs for treating neuropathic pain include antidepressants (serotonin–noradrenaline reuptake inhibitors and tricyclic antidepressants) and anticonvulsants, which are less effective for CIPN and accompanied by many unwanted side effects [5,13]. Therefore, there is an urgent need for safe and efficacious treatments for CIPN.

Increasing evidence indicates that neuropathic pain is correlated with chronic neuroinflammation caused by inflammatory factors [14]. Nociceptive dorsal root ganglion (DRG) neurons express proinflammatory cytokines and chemokine receptors that are upregulated in response to nerve injury, and proinflammatory molecules can sensitize nociceptors in C-fibers [15,16,17]. Neuroinflammation decreases the firing thresholds of A-δ and C-fiber nociceptors, leading to enhanced pain sensitivity [18]. Similarly, after nerve injury, infiltrating leukocytes release large amounts of proinflammatory cytokines to directly sensitize nociceptors, which in turn induce neuropathic pain [19]. Therefore, neuroinflammation and neuropathic pain can be partly reversed by inhibiting key proinflammatory cytokines and chemokines [20]. Nonsteroidal anti-inflammatory drugs (NSAIDs) are typically used for relieving inflammatory pain by inhibiting the enzyme cyclooxygenase (COX) and suppressing prostaglandin synthesis [21]. Moreover, NSAIDs have been widely used for the treatment of hyperalgesia, allodynia, and inflammation in both acute and chronic pain [22]. Pregabalin is one of the most commonly used analgesics to alleviate neuropathic pain, particularly CIPN, post-herpetic neuralgia, and diabetic neuropathy [13]. Previous studies have documented the neuroprotective effects of pregabalin in various pain models [23,24] and the anti-inflammatory properties in inflammatory pain models [25,26]. However, the potential role of NSAIDs as adjuvants for treating CIPN remains unclear. 

Previous studies have reported that coadministration of pregabalin and naproxen exerted a synergic antinociceptive effect on peripheral inflammation-associated thermal hyperalgesia [27], and coadministration of gabapentin and diclofenac also achieved significantly improved antihyperalgesic effects in a model of postoperative pain in rats [28]. Moreover, the combination of meloxicam and gabapentin showed enhanced antiallodynic and antihyperalgesic effects as compared with the drugs administered alone in a model of chronic constriction injury of the sciatic nerve in rats [29], and the combination of tramadol or pregabalin and ketorolac exhibited a marketed synergistic analgesic effect on mechanical and cold allodynia [30]. In a prospective and randomized controlled clinical trial, a combination of meloxicam and pregabalin was more effective in alleviating osteoarthritis pain (improved by approximately 10% to 25%) when compared with either treatment alone [31]. 

In view of the crosstalk between neuroinflammation and neuropathic pain, we investigated whether the synergistic interactions between NSAIDs (indomethacin or meloxicam) and pregabalin would extend to CIPN and inflammatory pain, thus providing therapeutic benefits for treating them. Specifically, we evaluated the antinociceptive effect of indomethacin, meloxicam, and pregabalin alone and their combinations in paclitaxel-induced neuropathic pain or carrageenan-induced inflammatory pain in rodents.

## 2. Materials and Methods

### 2.1. Animals

Adult male Institute of Cancer Research (ICR) mice (18–28 g) and adult male Sprague Dawley (SD) rats (230–280 g) were used as subjects for the experiments in this research project, and they came from Pizhou Dongfang Rabbit Breeding Co., Ltd. (Xuzhou, China). All animals were placed under standardized light, temperature, and humidity conditions with free access to standard feed and water. Animals were randomly assigned to different experimental groups, and mice or rats in each experimental group were housed in separate cages. Experiments were performed after a fasting period of at least 8 h. All studies involving animals in this study followed the guidelines of the Ethics and Experimental Animal Professional Committee of Jiangsu Ocean University (Approval code: 202100017).

### 2.2. Drugs and Treatment

Indomethacin, meloxicam, pregabalin, and paclitaxel were acquired from Shanghai Macklin Biochemical Co., Ltd. (Shanghai, China). λ-Carrageenan (the standard proinflammatory substance) [32] and sodium carboxymethyl cellulose were purchased from Shanghai Aladdin Biochemical Technology Co., Ltd. (Shanghai, China). Pregabalin was dissolved in saline, while indomethacin and meloxicam were suspended in a solution of 0.5% (*w*/*v*) sodium carboxymethyl cellulose. The drug was administered intragastrically (indomethacin and meloxicam) or intraperitoneally (pregabalin) in a volume of 0.1 mL/10 g for mice, with the vehicle group receiving the corresponding solvent (saline or 0.5% sodium carboxymethyl cellulose). All rats were administered via intraperitoneal or intragastric injection at a dose of 0.1 mL/100 g. λ-Carrageenan (2%, *w*/*v*) was prepared from normal saline. All solutions were prepared the same day the tests were performed. To identify the locus of the antinociceptive effect mediated by the combination of meloxicam and pregabalin in paclitaxel-induced neuropathic pain, meloxicam (5 μg/20 μL) or pregabalin (20 μg/20 μL) was injected intraplantarly into the surface of the left hind paw of mice.

### 2.3. Measurement of Antinociceptive Activity

#### 2.3.1. Paclitaxel-Induced Neuropathic Pain Model

Paclitaxel was administered intraperitoneally to induce painful peripheral neuropathy in mice [33]. With reference to Qabazard, B [34], paclitaxel was prepared with polyoxyethylene castor oil EL and ethanol at a concentration of 6 mg/mL and then diluted with normal saline to a final concentration of 0.2 mg/mL before administration. Similarly, the vehicle group was injected with saline diluted in the same proportion as the paclitaxel solution, which was composed of 1.67% polyoxyethylene castor oil EL and 1.67% saline ethanol. Paclitaxel (2 mg/kg) or its solvent was given to mice at a volume of 10 mL/kg (i.p.) once a day for 5 consecutive days. Mice were treated with indomethacin/meloxicam (p.o.), pregabalin (i.p.), or vehicle (p.o./i.p.) on day 7 when the mice developed mechanical allodynia. In order to evaluate the time course of the antiallodynic effect of each drug or combination, mechanical withdrawal thresholds were determined on day 7 post-paclitaxel, before (0 min), and 30, 60, 90, 120, 180, and 240 min after coadministration. The baseline mechanical thresholds were assessed 24 h before the induction of CIPN.

#### 2.3.2. Carrageenan-Induced Inflammatory Pain Model

The analgesic effect of indomethacin, meloxicam, pregabalin, and their combinations on mechanical allodynia was also evaluated in the carrageenan-induced inflammatory pain model [35,36]. In brief, the baseline values of rats responding to mechanical stimuli were assessed before intraplantar injection of 20 μL of 2% carrageenan in the left hind paws. Three hours after carrageenan injection, the rats were administered vehicle, indomethacin, meloxicam, pregabalin, and drug combinations. The mechanical paw withdrawal thresholds of the injured left hind paw in rats were measured before (0 min) and 30, 60, 90, 120, 180, and 240 min after administration. 

### 2.4. Assessment of Anti-Mechanical Allodynia Effect 

Mechanical allodynia was assessed using a dynamic plantar aesthesiometer (Anhui Zhenghua Biological Apparatus Facilities Co., Ltd., Huaibei, China). Briefly, mice or rats were left to acclimate for 30 min inside transparent enclosures on top of a perforated metal platform, and then a progressive force was imposed on the left hind paw (with a cut-off force of 50 g). The mechanical withdrawal threshold (MWT, expressed in grams) was automatically recorded when the animal removed its paw. Each test was measured three times and was performed at least 3 min after the last test to prevent injury.

The MWT of rodents was highest at 120 min after administration, so the percentage of the antinociception effect was calculated by the following formula.

% Antinociceptive Effect = (MWT of 120 min − MWT of 0 min)/(Baseline values − MWT of 0 min) × 100%

### 2.5. Data Analysis

#### 2.5.1. Isobolographic Analysis

Isobolographic analysis is widely accepted as the “gold standard” for the evaluation of pharmacological interactions (synergistic, additive, or antagonistic) between drugs in preclinical studies [37,38]. The interactions between indomethacin or meloxicam and pregabalin were characterized by isobolographic analysis, and the combinations comprised equieffective doses of the individual component drugs [39]. Considering that indomethacin was incapable of exerting 50% suppression of mechanical allodynia, it was not feasible to calculate ED_50_ for the antiallodynic effect. Therefore, we determined the antinociceptive effect of ED_25_ instead of ED_50_. The theoretical ED_25_ values of the combinations were calculated from each dose–response curve, and the combinations were considered the sum of the effects of each drug [37]. Subsequently, a dose–response curve was obtained by coadministration of two drugs (indomethacin or meloxicam plus pregabalin) in a fixed ratio of proportions of the equieffective ED_25_ doses for each drug alone. Each group of mice or rats received the following combined doses: (1) 2 × (IN/MEL ED_25_ + PGB ED_25_); (2) IN/MEL ED_25_+ PGB ED_25_; (3) (IN/MEL ED_25_ + PGB ED_25_)/2; (4) (IN/MEL ED_25_ + PGB ED_25_)/4; and (5) (IN/MEL ED_25_ + PGB ED_25_)/8, as shown in Table 1 and Table 2. The experimental ED_25_ (ED_25_E) was calculated from the response curve of the IN-PGB or MEL-PGB combination. The isobologram was illustrated by connecting the ED_25_ of the indomethacin or meloxicam on the abscissa with the ED_25_ of the corresponding pregabalin on the ordinate to obtain the additivity line [40]. To determine the pharmacological interaction of indomethacin or meloxicam with pregabalin (synergism, γ < 1; additive, γ = 1; antagonism, γ > 1), the value of ED_25_T (the theoretical ED_25_) was compared with the combined experimental ED_25_E [37]. The interaction index (γ) was calculated with the following formula:

$$\gamma =\frac{{\mathrm{ED}}_{25}\;\mathrm{of}\;\mathrm{the}\;\mathrm{combination}\;(\mathrm{experimental})}{{\mathrm{ED}}_{25}\;\mathrm{of}\;\mathrm{the}\;\mathrm{combination}\;(\mathrm{theoretical})}$$

#### 2.5.2. Statistical Analysis

Data were analyzed by one-way or two-way analysis of variance (ANOVA). We used GraphPad Prism (GraphPad Software Inc., San Diego, CA, USA) to calculate the area under the curve (AUC) in the time course study. We checked the normality of the distribution of the results by the Shapiro–Wilk test. The normality test indicated the normal distribution of the AUC data, and Dunnett’s test was applied for post hoc analysis of the AUC. For the dose–response study and analysis, we used two-way ANOVA followed by Tukey’s post hoc. All data are expressed as mean ± S.E.M. For all statistical analyses, *p* < 0.05 was considered to be statistically significant. GraphPad Prism version 7.0 (GraphPad Software Inc.) was employed for all statistical analyses.

## 3. Results

### 3.1. The Synergistic Antinociceptive Effects of the Combination Treatment of Indomethacin and Pregabalin on Paclitaxel-Induced Neuropathic Pain

**Figure 1:**
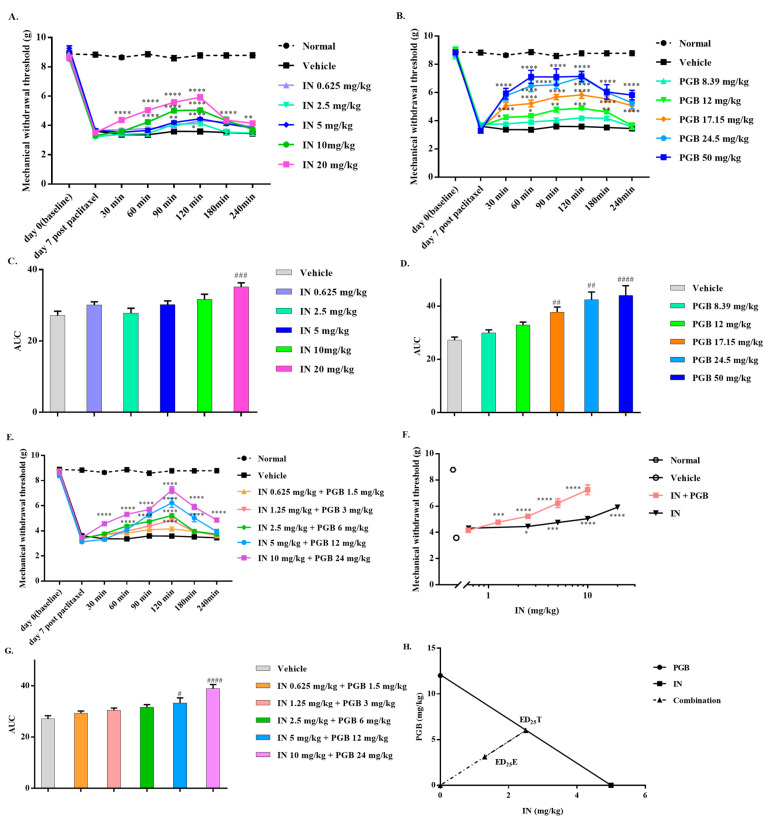
Effect of indomethacin (IN), pregabalin (PGB), and IN-PGB on paclitaxel-induced neuropathic pain in mice. Time course of the antiallodynic effects evoked by IN (**A**) and PGB (**B**). The AUC of IN or PGB indicates that they reversed paclitaxel-induced mechanical allodynia in a dose-related manner (**C**,**D**). Equieffective doses of IN and PGB were administered in combination to reverse mechanical allodynia (**E**). An equieffective combination of PGB and IN made the dose–response curve shift to the left (**F**). Time course effects of the IN-PGB are expressed as the AUC (**G**). The equieffective combination of IN and PGB produced a synergistic effect, as ED_25_E fell below the line of additivity (**H**). For this graph, the derived ED_25_ value of IN is plotted on the abscissa, and the ED_25_ value of PGB is plotted on the ordinate. * *p* < 0.05, ** *p* < 0.01, *** *p* < 0.001, and **** *p* < 0.0001 versus vehicle by two-way ANOVA followed by Tukey’s test. ^#^ *p* < 0.05, ^##^ *p* < 0.01, ^###^ *p* < 0.001, and ^####^ *p* < 0.0001 versus vehicle by one-way ANOVA followed by Dunnett’s test. Data are expressed as mean ± S.E.M., n = 8/group.

To evaluate the antiallodynic effect of drugs, the MWTs were determined on day 7 post-paclitaxel, before (0 min), and 30, 60, 90, 120, 180, and 240 min after administration of the drugs. On day 7, the MWTs of paclitaxel-injected mice were reduced as compared with the baseline values, indicating that our animal model construction was successful. Indomethacin (IN) dose-dependently reversed mechanical allodynia in paclitaxel-injected mice, with a peak effect at 120 min after administration (Figure 1A). Pregabalin (PGB) effectively reversed the mechanical allodynia induced by paclitaxel in a dose-dependent manner in mice, and the peak effect occurred 60 min after administration, which was maintained until 120 min (Figure 1B). Indomethacin and pregabalin had respective ED_25_ values (95% confidence limits) of 5.00 (3.68–6.23) and 12.01 (9.57–14.41) mg/kg. Figure 1C,D illustrate the antiallodynic effects evoked by indomethacin and pregabalin over time, expressed as area under the curve (AUC). AUC data show that indomethacin or pregabalin dose-dependently reversed paclitaxel-induced mechanical allodynia. Coadministration of indomethacin and pregabalin can reverse the mechanical allodynia induced by paclitaxel in a dose-dependent manner (Figure 1E,G). The combined administration of indomethacin and pregabalin produced a leftward shift in the dose–response curve compared to indomethacin alone (Figure 1F). In Figure 1H, the isobolographic analysis shows that there was a synergistic effect between these drugs. The calculated experimental ED_25_E value [4.41 (3.13–5.82)] was significantly lower than the calculated theoretical ED_25_T value [8.50 (6.62–10.32)] and below the line of additivity (γ = 0.52).

### 3.2. The Synergistic Antinociceptive Effects of the Combination Treatment of Meloxicam and Pregabalin on Paclitaxel-Induced Neuropathic Pain

**Figure 2:**
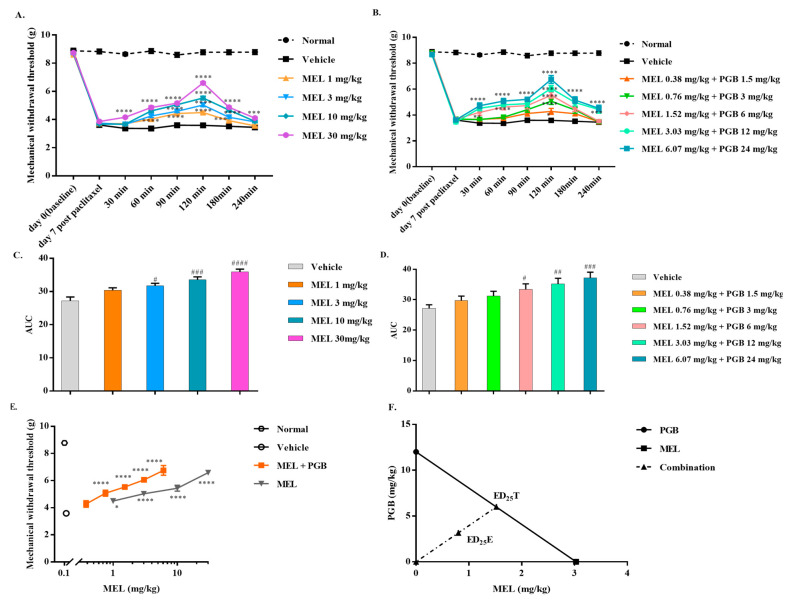
Effect of meloxicam (MEL), pregabalin (PGB), and MEL-PGB on paclitaxel-induced neuropathic pain in mice. Time course of the antiallodynic effects evoked by MEL and the combination of MEL and PGB (**A**,**B**). The AUC of MEL or MEL-PGB indicates that they reversed paclitaxel-induced mechanical allodynia in a dose-related manner (**C**,**D**). Equieffective doses of MEL and PGB were administered in combination to reverse mechanical allodynia in a dose-dependent manner (**E**). An equieffective combination of PGB and MEL made the dose–response curve shift to the left (**F**). Isobolographic analysis supported a synergistic analgesic effect of MEL-PGB on paclitaxel-induced mechanical allodynia (**F**). * *p* < 0.05, ** *p* < 0.01, *** *p* < 0.001, and **** *p* < 0.0001 versus vehicle by two-way ANOVA followed by Tukey’s test. ^#^ *p* < 0.05, ^##^ *p* < 0.01, ^###^ *p* < 0.001, and ^####^ *p* < 0.0001 versus vehicle by one-way ANOVA followed by Dunnett’s test. Bars are the mean ± S.E.M. of eight animals.

Experimental procedures for the administration of meloxicam in combination with pregabalin are described in Section 2.5.1. The mechanical allodynia induced by paclitaxel (Figure 2A) was dose-dependently reduced by meloxicam, with an ED_25_ value of 3.03 (1.90–4.38) mg/kg. Coadministration of meloxicam and pregabalin could also reverse the mechanical allodynia induced by paclitaxel in a dose-dependent manner (Figure 2B). The increased maximal effect for the AUC data in the combinations compared with either meloxicam or pregabalin alone is in accordance with the time course of the antiallodynic effects evoked by the combination of meloxicam and pregabalin (Figure 1D and Figure 2C,D). MEL and MEL-PGB reversed allodynia in a dose-related manner, with a peak effect at 120 min after administration. The dose–response curve of the combination of meloxicam and pregabalin shifted to the left after coadministration compared with meloxicam alone (Figure 2E). As shown in Figure 2F, the thick oblique line represents the theoretical additive line. The point “ED_25_T” in the middle of the line is the theoretical additive point calculated from the individual drug ED_25_ values. The experimental point indicated by “ED_25_E” is the actual ED_25_ value for that combination. The isobolographic analysis indicated a synergistic effect, which can be inferred from the experimental ED_25_E values [3.96 (2.62–5.46)] below the additive line (ED_25_T = [7.52 (5.73–9.39)], γ = 0.53).

### 3.3. The Locus of the Antinociceptive Effect Mediated by the Combination of Meloxicam and Pregabalin in Paclitaxel-Induced Neuropathic Pain

**Figure 3:**
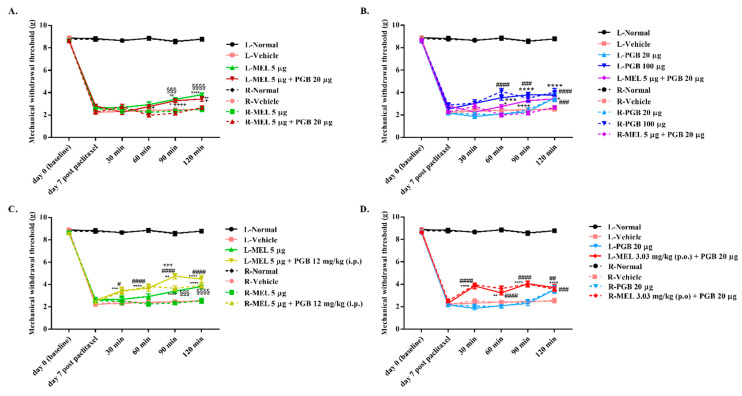
Antinociceptive effect of intraplantar or systemic administration of MEL, PGB, and MEL-PGB on paclitaxel-induced neuropathic pain in mice. Time course effects on the MWTs produced by intraplantar administration of MEL, PGB, and MEL-PGB in mice (**A**,**B**). The analgesic effect of combined administration of MEL and PGB was more significant than monotherapy with intraplantar administration of MEL or PGB (**C**,**D**). L and R represent the left and right hind paws of the mice, respectively. * *p* < 0.05, ** *p* < 0.01, *** *p* < 0.001, and **** *p* < 0.0001 versus L-vehicle by two-way ANOVA followed by Tukey’s test. ^#^ *p* < 0.05, ^##^ *p* < 0.01, ^###^ *p* < 0.001, and ^####^ *p* < 0.0001 versus R-vehicle by two-way ANOVA followed by Tukey’s test. ^§§§^ *p* < 0.001, ^§§§§^ *p* < 0.0001, ^+^ *p* < 0.05, ^+++^ *p* < 0.001, and ^++++^ *p* < 0.0001 versus the MWTs of the contralateral paw of mice by two-way ANOVA followed by Tukey’s test. Data are expressed as mean ± S.E.M., n = 8/group.

Intraplantar administration of meloxicam (5 μg) into the left paw produced a significant analgesic effect (* *p* < 0.05) and was significantly different from the MWTs of the contralateral paw (^§^ *p* < 0.05), which excludes the possibility of the antiallodynic effects occurring because of diffusion from the injection site (Figure 3A). Pregabalin (100 μg, intraplantar injection to the left paw) also reversed paclitaxel-induced allodynia in a time-dependent manner and was not different from the MWTs of the contralateral paw, whereas pregabalin (20 μg) reversed mechanical allodynia in paclitaxel-injected mice with a significant effect only at 120 min after administration. Coadministration of meloxicam (5 μg) and pregabalin (20 μg) significantly reversed paclitaxel-induced allodynia, which is similar to meloxicam (5 μg) given alone but significantly greater than pregabalin (20 μg) alone (Figure 3B). On the other hand, the combination of meloxicam (5 μg) with pregabalin (12 mg/kg, i.p.) significantly inhibited mechanical allodynia in paclitaxel-treated mice, and there was a significant difference between the MWTs of the left and right hind paws starting at 90 min (^§^ *p* < 0.05, ^+^ *p* < 0.05) (Figure 3C). Similarly, coadministration of meloxicam (3.03 mg/kg, p.o.) with pregabalin (20 μg) significantly alleviated mechanical allodynia in paclitaxel-treated mice (Figure 3D).

### 3.4. The Antagonistic Antinociceptive Effects of the Combination Treatment of Indomethacin and Pregabalin in Carrageenan-Induced Inflammatory Pain in Rats

**Figure 4:**
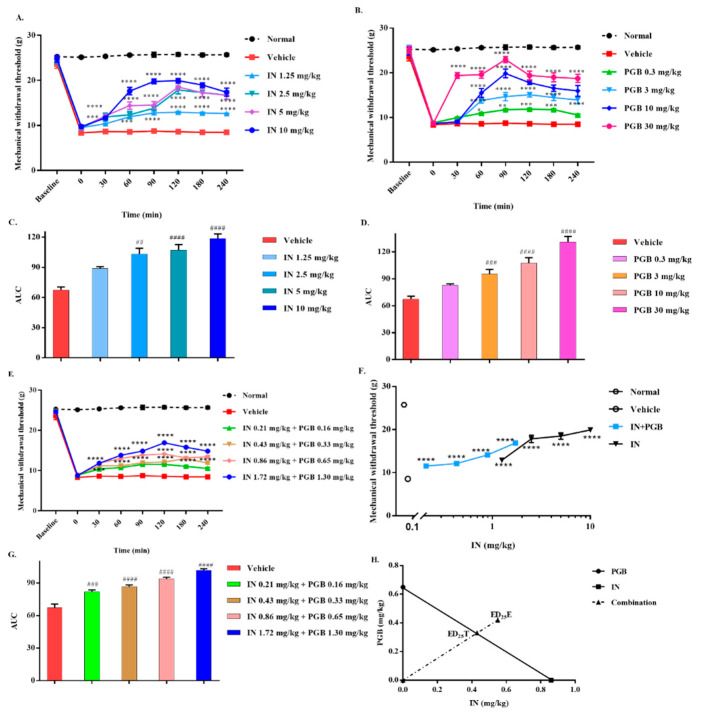
Effect of IN, PGB, and IN-PGB on carrageenan-induced mechanical allodynia. Time course effects on the MWT produced by administration of IN (**A**) or PGB (**B**) in rats. The AUC shows that an injection of IN, PGB, or IN-PGB reversed carrageenan-induced allodynia in a dose-related manner (**C**,**D**). Equieffective doses of IN and PGB were administered in combination to reverse mechanical allodynia (**E**). The combination of PGB and IN made the dose–response curve move to the left (**F**). Time course effects of IN-PGB are expressed as the AUC (**G**). Isobolographic analysis indicated an antagonistic/subadditive interaction between IN and PGB on carrageenan-induced mechanical allodynia (**H**). * *p* < 0.05, ** *p* < 0.01, *** *p* < 0.001, and **** *p* < 0.0001 versus vehicle by two-way ANOVA followed by Tukey’s test. ^##^ *p* < 0.01, ^###^ *p* < 0.001, and ^####^ *p* < 0.0001 versus vehicle by one-way ANOVA followed by Dunnett’s test. Data are expressed as mean ± S.E.M., n = 6 rats/group.

After carrageenan was injected, the paw surface of the rat gradually became red and swollen, the paw volume increased, and the rat gradually curled up its head and slightly lifted the injured left hind paw with time. The sensitivity of the injured left hind paw to mechanical stimulation increased, and the change in the MWT after indomethacin or pregabalin administration is shown in Figure 4A,B. Indomethacin and pregabalin produced statistically significantly higher MWTs in carrageenan-induced mechanical allodynia and are expressed as AUC (Figure 4C,D). 

The ED_25_ values for indomethacin and pregabalin in carrageenan-induced allodynia in rats were [0.86 (0.45–1.35)] mg/kg and [0.65 (0.31–1.17)] mg/kg, respectively. The procedure of combined administration is described in Section 2.5.1, and the dosage of the drug is shown in Table 2. The combination of indomethacin and pregabalin exhibited a dose-dependent antiallodynic effect in this model (Figure 4E,G). The combination of equieffective doses of indomethacin and pregabalin produced a leftward shift in the dose–response curve compared with that of indomethacin alone (Figure 4F). However, the experimental ED_25_E value [0.96 (0.89–1.02)] was significantly higher than the theoretical ED_25_T value [0.76 (0.38–1.26)] and above the line of additivity (Figure 4H). The interaction index (γ) for the indomethacin–pregabalin combination was 1.25, which indicates that there was a subadditive interaction.

### 3.5. The Antagonistic Antinociceptive Effects of the Combination Treatment of Meloxicam and Pregabalin in Carrageenan-Induced Inflammatory Pain in Rats 

**Figure 5:**
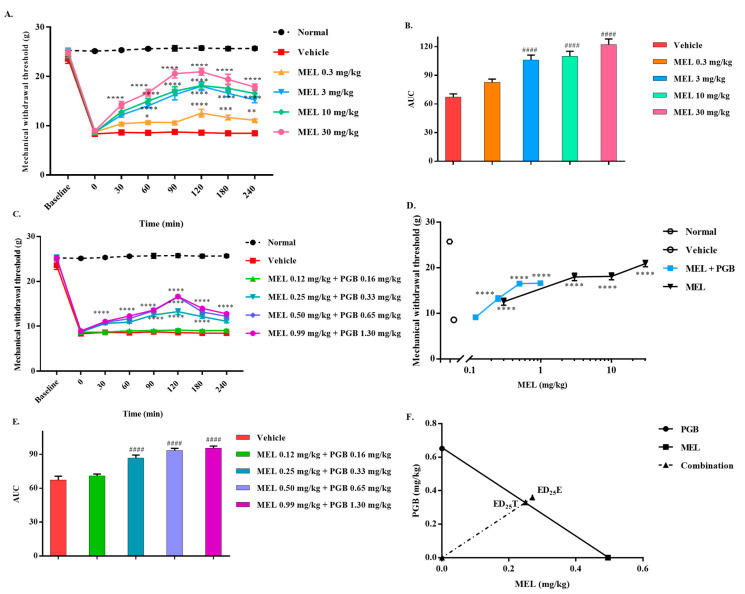
Effect of MEL, PGB, and MEL-PGB on carrageenan-induced mechanical allodynia. Time course effects on the MWT produced by administration of MEL in rats (**A**). The AUC data show that MEL or MEL-PGB reversed carrageenan-induced allodynia in a dose-related manner (**B**). Time course of the antiallodynic effects evoked by MEL-PGB administration in rats (**C**). The combination of PGB and MEL made the dose–response curve move to the left (**D**). Equieffective doses of IN and PGB were administered in combination to reverse mechanical allodynia (**E**). Isobolographic analysis indicated an antagonistic/subadditive interaction between MEL and PGB on carrageenan-induced mechanical allodynia (**F**). * *p* < 0.05, ** *p* < 0.01, *** p < 0.001, and **** *p* < 0.0001 versus vehicle by two-way ANOVA followed by Tukey’s test. ^####^ *p* < 0.0001 versus vehicle by one-way ANOVA followed by Dunnett’s test. Data are expressed as mean ± S.E.M., n = 6 rats/group.

Carrageenan-induced inflammatory pain in rats was reversed dose-dependently by meloxicam (Figure 5A,B). Equieffective doses of meloxicam and pregabalin given in combination effectively reversed the mechanical allodynia induced by carrageenan (Figure 5C). Compared to meloxicam alone, the antinociceptive effect was not significantly enhanced, despite a leftward shift in the dose–response curve following the combination of meloxicam and pregabalin (Figure 5D,E). After analysis of the data, ED_25_E [0.63 (0.44–0.82)] was higher than ED_25_T [0.58 (0.26–1.1)] after coadministration of meloxicam and pregabalin, which means that ED_25_E is above the equivalence line, and therefore, γ > 1 (γ = 1.09, Figure 5F). These results suggest that although meloxicam and pregabalin can alleviate carrageenan-induced mechanical allodynia in rats, there may be antagonistic (subadditive) interactions between the two drugs in this model.

## 4. Discussion

CIPN reflects the major dose-limiting neurotoxicity associated with chemotherapeutic agents and may last for years, which greatly compromises cancer patients’ quality of life due to the lack of safe and efficacious treatments with available analgesics [41]. The current first-line drugs (anticonvulsants, tricyclic antidepressants, and serotonin and norepinephrine reuptake inhibitors [42,43]) for treating neuropathic pain often cause adverse reactions, including hypertension, drowsiness, dizziness, and severe headache in patients [44]. Among them, tricyclic antidepressants are even associated with tachycardia and myocardial infarction and have limited analgesic effects [45]. Combinations of available analgesics (multimodal analgesia) represent a practical strategy to achieve higher antinociceptive efficacy and fewer side effects than monotherapy because the synergistic effects between different drugs enable a reduction in the dose and thus a decrease in the incidence of side effects [46].

The present study assessed whether coadministration of indomethacin/meloxicam with pregabalin would more effectively alleviate paclitaxel-induced allodynia in mice. The results showed that indomethacin, meloxicam, and pregabalin dose-dependently reversed mechanical allodynia in paclitaxel-treated mice. Isobolographic analysis was conducted to characterize the interaction of indomethacin or meloxicam with pregabalin in CIPN. Strikingly, the combined treatment of indomethacin/meloxicam and pregabalin exerted synergistic antiallodynic effects in paclitaxel-treated mice, with a more pronounced antinociceptive effect compared with the single administration of these drugs. The initiation and maintenance of neuropathic pain are closely associated with excessive inflammation in both the peripheral and central nervous systems [47]. Baba et al. reported that NSAIDs cause presynaptic neurons to inhibit glutamate release and reduce the excitability of postsynaptic dorsal horn neurons through the inhibition of PGE_2_ synthesis [48]. Pregabalin strongly binds to the α_2_-δ subunit of the Ca^2+^ channel and may decrease Ca^2+^ influx through the presynaptic inhibition of glutamate release [27]. The above signal cascades may partly explain the synergistic analgesic effect between NSAIDs and calcium channel modulators in CIPN. 

In addition, we found that indomethacin or meloxicam and pregabalin combined administration produced antagonistic antiallodynic effects in carrageenan-induced inflammatory pain in rats, although they had a more pronounced analgesic effect than when administered alone. Carrageenan is a known proinflammatory substance that induces hyperalgesia and allodynia in rodents [49]. Carrageenan-stimulated acute inflammation pain is characterized by a biphasic pain response. The initial phase (0–2 h) is primarily induced by the rapid production of inflammatory mediators and increased synthesis of prostaglandins in the damaged tissues, and the late phase (3–6 h) is sustained by prostaglandin release from macrophages [50]. Hence, NSAIDs can exert a pronounced antiallodynic effect through the inhibition of PGE_2_ synthesis on carrageenan-induced inflammatory pain. However, the underlying mechanism for the antagonistic interaction between NSAIDs and pregabalin in this model remains to be solved.

To identify the locus of action of the antiallodynic effects of MEL-PGB, we examined whether MEL, PGB, or MEL-PGB would reverse paclitaxel-induced allodynia when given via intraplantar injection into the left hind paw or via intragastric or intraperitoneal administration. Our results demonstrated that intraplantar injection of meloxicam (5 µg) reversed allodynia in the injected paw but not in the contralateral paw, supporting a peripheral site of action. When pregabalin (20 μg) was intraplantarly injected into the left paw, the contralateral paw also showed an antinociceptive effect at 120 min, indicating that pregabalin first diffused from the injection site into the central nervous system and then exerted its antiallodynic effects in the neuroaxis. Additionally, coadministration of meloxicam (5 μg) with pregabalin (12 mg/kg, i.p.) enhanced the analgesic effect of meloxicam (5 μg). Similarly, the combination of meloxicam (3.03 mg/kg, p.o.) with pregabalin (20 μg) enhanced the analgesic effect of pregabalin (20 μg) in paclitaxel-induced neuropathic pain, suggesting that both peripheral and central sites of action are involved. According to reported references, pregabalin acts supraspinally through the descending noradrenergic pain inhibitory system coupled with spinal α_2_-adrenergic receptors to produce antinociceptive effects after peripheral nerve injury [51]; NSAIDs act upon both the peripheral tissues and the central nervous system to exert analgesia [52]; and paclitaxel-induced mechanical hypersensitivity is due to central sensitization that abnormally produces pain in response to Aβ fiber inputs [53]. These findings indicate that the antiallodynic effects of MEL-PGB on paclitaxel-induced neuropathic pain can act at multiple sites in the neuroaxis and periphery.

Neuroinflammation can contribute to the development of CIPN due to the activation of the immune system by chemotherapeutics that may lead to neuroinflammation [54]. Numerous studies have indicated that raised levels of proinflammatory cytokines sensitize peripheral sensory neurons, leading to CIPN, particularly when found in the dorsal root ganglia or spinal cord [55]. Peripheral cytokines that entered the central nervous system (CNS) added to the neuroinflammatory response when the permeability of the blood–brain barrier increased [56]. On the other hand, chemotherapy-induced changes in the CNS have also been implicated in CIPN. In the CNS, paclitaxel induced significant astrocyte activation in the spinal cord dorsal horn and caused a reduction in P2ry12+ homeostatic microglia in the dorsal and ventral horns [57]. Taken together, the pathological changes in both the periphery and nervous system may contribute to CIPN. Among other neuropathic pain conditions, trigeminal neuralgia has been shown to frequently cooccur with the demyelinating neuroinflammatory disease multiple sclerosis [58]. Ericson et al. believe that abnormal inflammatory mechanisms are also involved in the pathophysiology of trigeminal neuralgia [59]. Moreover, studies have found that 30–50% of patients with vasculitis have symptoms of peripheral neuropathy [60]. It can be seen that there is an inextricable relationship between neuropathic pain and inflammation. The efficacy of pregabalin and NSAIDs in the treatment of neuroinflammation and related neuropsychiatric disorders has also been reported. Eman et al. evidenced the anti-inflammatory properties of pregabalin on cytokine secretion and lymphoid organ inflammation [25], and pregabalin prevents neuroinflammation during the early postoperative period through a peripheral and central neuro-immune interaction [61]. NSAIDs can significantly reduce the expression of COX-2 and PGE**_2_** and the infiltration of macrophages, resulting in alleviated neuroinflammation in chronic constriction injury rats [62]. Therefore, NSAIDs may serve as adjuvants for treating neuropathic pain, including CIPN, trigeminal neuralgia, etc. 

## 5. Conclusions

In conclusion, we showed that coadministration of indomethacin or meloxicam and pregabalin has synergistic analgesic effects in paclitaxel-induced neuropathic pain, and low doses of each drug in combination can exert significant reductions in mechanical allodynia, thereby minimizing the risk of side effects from their longer-term use. However, the therapeutic efficacy of the combinations of indomethacin or meloxicam with pregabalin is not significantly improved in carrageenan-induced inflammatory pain. Our results indicate that combination therapies between pregabalin and NSAIDs such as indomethacin or meloxicam may possess potential therapeutic advantages for relieving chemotherapy-induced neuropathic pain, particularly among the elderly population at increased risk for the adverse effects of NSAIDs. We offer an alternative therapeutic strategy for neuropathic pain patients. These combination therapies may synergize the antinociceptive effects of these drugs and provide several advantages, including better efficacy, as well as dose reduction of the individual agents involved to minimize their adverse effects. While these benefits appear attractive, further exploration is required to study the underlying mechanisms of these combinations in in vitro and in vivo assays, which is the next step in our research.

## Figures and Tables

**Table 1:** Doses of indomethacin or meloxicam combined with pregabalin in paclitaxel-induced neuropathic pain in mice.

Groups (n = 8)	Dose (mg/kg)		Antinociceptive Effect (%) ^a^
	Indomethacin	Pregabalin	Combination
(1)	10.00	24	72.69 ± 7.17
(2)	5.00	12	58.89 ± 6.40
(3)	2.50	6	35.50 ± 1.74
(4)	1.25	3	25.96 ± 2.51
(5)	0.63	1.5	14.61 ± 3.61
	Meloxicam	Pregabalin	Combination
(1)	6.07	24	61.98 ± 7.02
(2)	3.03	12	50.04 ± 3.16
(3)	1.52	6	36.13 ± 2.56
(4)	0.76	3	27.10 ± 4.34
(5)	0.38	1.5	11.87 ± 4.77

**^a^** The antinociceptive effect expressed in percentage was obtained from the formula mentioned above (data expressed as mean ± S.E.M., n = 8 mice/group).

**Table 2:** Dosages of indomethacin or meloxicam combined with pregabalin in carrageenan-induced inflammatory pain in rats.

Groups (n = 6)	Dose (mg/kg)		Antinociceptive Effect (%) ^b^
	Indomethacin	Pregabalin	Combination
(1)	1.72	1.30	51.14 ± 1.14
(2)	0.86	0.65	33.66 ± 0.58
(3)	0.43	0.33	20.64 ± 0.75
(4)	0.21	0.16	16.92 ± 1.45
	Meloxicam	Pregabalin	Combination
(1)	0.12	1.30	47.25 ± 0.91
(2)	0.25	0.65	47.14 ± 1.00
(3)	0.50	0.33	27.23 ± 2.10
(4)	0.99	0.16	2.78 ± 4.43

^b^ The antinociceptive effect expressed in percentage was obtained from the formula mentioned above (data expressed as mean ± S.E.M., n = 6 rats/group).

## Data Availability

Not applicable.
